# From 1D to 2D Cd(II) and Zn(II) Coordination Networks by Replacing Monocarboxylate with Dicarboxylates in Partnership with Azine Ligands: Synthesis, Crystal Structures, Inclusion, and Emission Properties

**DOI:** 10.3390/molecules25235616

**Published:** 2020-11-29

**Authors:** Victor Ch. Kravtsov, Vasile Lozovan, Nikita Siminel, Eduard B. Coropceanu, Olga V. Kulikova, Natalia V. Costriucova, Marina S. Fonari

**Affiliations:** 1 Institute of Applied Physics, Academiei 5, MD2028 Chisinau, Moldova; kravtsov@phys.asm.md (V.C.K.); siminel.n@gmail.com (N.S.); olga.kulikova@phys.asm.md (O.V.K.); constriucova@phys.asm.md (N.V.C.); 2 Institute of Chemistry, Academiei 3, MD2028 Chisinau, Moldova; lozovanvasile90@gmail.com (V.L.); coropceanu.eduard@ust.md (E.B.C.); 3 Chemistry Department, Tiraspol State University, Iablocikin 5, MD2069 Chisinau, Moldova

**Keywords:** carboxylate ligands, azine ligands, coordination polymers, crystal structure, spectroscopic studies, solvent exchange, emission properties

## Abstract

Eight mixed-ligand coordination networks, [Cd(2-aba)(NO_3_)(4-bphz)_3/2_]_n_·n(dmf) (**1**), [Cd(2-aba)_2_(4-bphz)]_n_·0.75n(dmf) (**2**), [Cd(seb)(4-bphz)]_n_·n(H_2_O) (**3**), [Cd(seb)(4-bpmhz)]_n_·n(H_2_O) (**4**), [Cd(hpa)(3-bphz)]_n_ (**5**), [Zn(1,3-bdc)(3-bpmhz)]_n_·n(MeOH) (**6**), [Cd(1,3-bdc)(3-bpmhz)]_n_ ·0.5n(H_2_O)·0.5n(EtOH) (**7**), and [Cd(NO_3_)_2_(3-bphz)(bpe)]_n_·n(3-bphz) (**8**) were obtained by interplay of cadmium nitrate tetrahydrate or zinc nitrate hexahydrate with 2-aminobenzenecarboxylic acid (H(2-aba)), three dicarboxylic acids, sebacic (decanedioic acid, H_2_seb), homophthalic (2-(carboxymethyl)benzoic acid, H_2_hpa), isophthalic (1,3-benzenedicarboxylic acid, H_2_(1,3-bdc)) acids, bis(4-pyridyl)ethane (bpe) and with four azine ligands, 1,2-bis(pyridin-4-ylmethylene)hydrazine (4-bphz), 1,2-bis(1-(pyridin-4-yl)ethylidene) hydrazine (4-bpmhz), 1,2-bis(pyridin-3-ylmethylene)hydrazine (3-bphz), and 1,2-bis(1-(pyridin-3-yl) ethylidene)hydrazine (3-bpmhz). Compounds **1** and **2** are 1D coordination polymers, while compounds **3**–**8** are 2D coordination polymers. All compounds were characterized by spectroscopic and X-ray diffraction methods of analysis. The solvent uptakes and stabilities to the guest evacuation were studied and compared for 1D and 2D coordination networks. The de-solvated forms revealed a significant increase of emission in comparison with the as-synthesized crystals.

## 1. Introduction

Having been launched as intriguing adsorption materials with the possibility to be applied in gas storage due to the exceptional low density, large surface area, pore function regulation, and structural flexibility [1,2], currently, the coordination polymers (CP) demonstrate their significance as materials for luminescence [3], catalysis [4], magnetism [5], optics [6], electric conductivity [7], and so on. The pore regulation by design can be considered as an advantage of CPs compared to the traditional porous materials such as zeolites and activated carbons. The guest species like solvents, ions, or confined organic molecules accommodated in the pores can act as templates and determine the shapes and sizes of pores [8,9]. Alongside the rigid 3D Metal-Organic Frameworks (MOFs), impressive adsorption properties accompanied by the guest selectivity were reported for 1D and 2D coordination networks [10,11,12].

The carboxylates are among the most demanded ligands in the synthesis of homo-ligand and hetero-ligand CPs with either rigid or flexible coordination backbones [13]. The rational choice of the metal provides the ability of the carboxylic group to coordinate to the metal in different ways and ensures the formation of coordination networks with different dimensionality and complexity [14,15,16]. The carboxylate ligands afford an access to the neutral metal-organic carcasses due to the direct cation-anion coordination bonds, compared to the cationic networks with the neutral ligands, where the anions occupy the voids in the polymeric network. The increase in the number of ligand donor atoms accompanied by the increase in the number of chelate rings usually leads to greater stability of the complexes [17,18].

Schiff-bases that possess a spectrum of useful properties [19] are widely used as carboxylates′ co-ligands in coordination polymers [20]. Several groups showed how manipulating by rigidity of both dicarboxylates and N, N′-donor ligands provided an access to different Zn(II) and Cd(II) 2D and 3D coordination networks. The 2D interdigitated structures revealed a change not only the void volume in the framework, but also the flexibility of structures, the chemical affinity, and effective pore shape and selectivity in guest sorption [21,22,23,24,25].

Recently, we reported a series of 1D Zn(II)/Cd(II) CPs comprising monocarboxylic 2-thiophenecarboxylic acid and different N, N′-azine ligands where the criss-cross packing mode of rigid coordination chains facilitated an access to the significant amount of space in the crystal structures predisposed to the guest accommodation and exchange [26].

Herein, we report our study on the influence of synthetic conditions on the resulting structure topology and dimensionality of eight Cd/Zn CPs comprising different azine and carboxylate ligands and their solvent exchange and emission properties. We have demonstrated that replacement of monocarboxylate by dicarboxylate anions allowed extending the dimensionality of coordination polymers from 1D to 2D, keeping invariable the coordination polymeric chains originating from azine ligands.

## 2. Results and Discussion

### 2.1. Generals

**Scheme 1:**
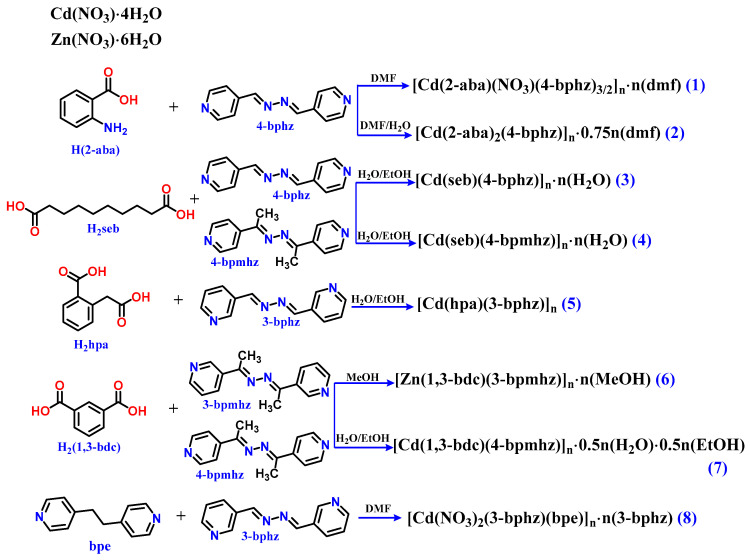
Schematic pathway for coordination polymers **1**–**8**.

**Figure 1:**
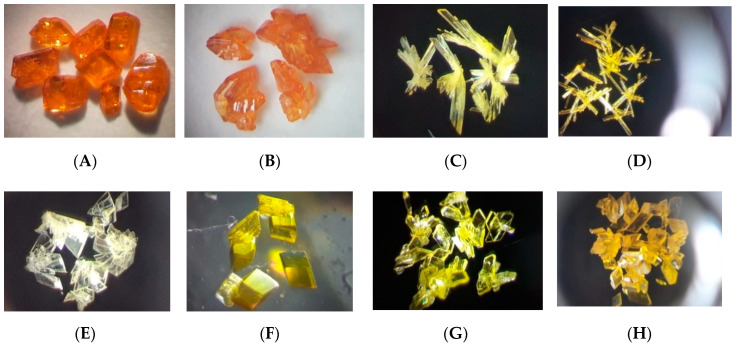
Crystal images for compounds **1**–**8** (**A**–**H**).

The reported CPs **1**–**8** (Scheme 1) were obtained by slow evaporation of the solvent or slow diffusion method, either in the glass beaker or in the test tube, starting from the solution Zn(II) or Cd(II) nitrate salts, mono-/dicarboxylic acid, and one of four azomethine ligands or by using two bipyridine-type ligands in the case of **8**. The crystal images for **1**–**8** are shown in Figure 1. Crystal colors vary from yellow to orange-red.

### 2.2. Description of Crystal Structures

#### 2.2.1. 1D Coordination Polymers

**Figure 2:**
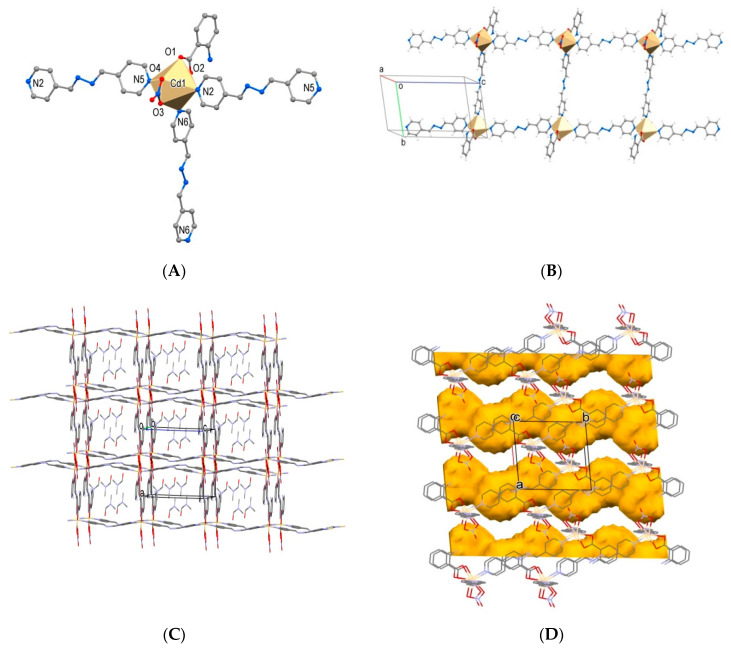
(**A**) Cd(II) coordination core in **1**. (**B**) Fragment of coordination chain in **1** with indication of unit cell. (**C**) Fragment of crystal packing with dmf accommodation. (**D**) Wave-like SAVs in **1** shown in yellow. View along *c* axis. H-atoms are omitted.

Two 1D coordination polymers [Cd(2-aba)(NO_3_)(4-bphz)_3/2_]_n_·n(dmf) (**1**), and [Cd(2-aba)_2_(4-bphz)]_n_·0.75 n(dmf) (**2**), were obtained from the same solution crystallization without heating for **1** and with gentle heating for **2**. The latter procedure has resulted in complete substitution of a starting nitrate anion in the final product. Compound **1** crystallizes in the form of a ladder-type coordination chain with the Cd(II) atom as a single metal node. The asymmetric unit comprises one Cd(II) cation, one nitrate, and one aba anions, and one and a half 4-bphz ligands. The Cd(II) atom distorted pentagonal-bipyramidal N_3_O_4_ coordination geometry, which is going from one nitrate and one 2-aba anion along with one 4-bphz ligand in the basal plane, and two 4-bphz ligands situated in *trans*-axial positions (Figure 2A). Both anions are the terminal ones and coordinate to the metal in the bidentate chelate coordination mode (µ_1_-η^2^). All 4-bphz ligands take the bidentate-bridging modes of coordination, providing possibilities for structure extension. Thus, a coordination polyhedron of the metal may be viewed as a T-shaped three-connected node, and resulted in the ladder-like coordination chain, running along the *c* crystallographic axis (Figure 2B). All 4-bphz ligands are in *trans*-conformations, thus, providing similar Cd···Cd separations of 15.937(1) and 16.131(1) Å along and across the ladder. The chains pack in the *bc* plane in the interdigitated mode with the parallel arrangement of aromatic pyridine rings of the 2-aba residues and 4-bphz ligands with Cg···Cg separation of 4.322 Å. Such packing provides wave-like channels filled by the dmf molecules. The dmf solvent is held in the channels via H(CH_3_)···O(NO_3_) and CH(4-bphz)···O(CO) hydrogen bonds, C(13)-H(13)···O(1S)(1 − x, 1 − y, 1 − z) (2.45 3.276(13) Å, ∠CHO 148°), C(3S)-H(3S2)···O(5) (2.53, 3.118(18) Å, ∠CHO 120°) (Figure 2C,D). The solvent-accessible voids (SAVs) calculated with the guest removal from the structure comprise 23% or 375.7 Å^3^ of the unit cell volume. The Cd(II) coordination core in **1** is identical with that one in 1D CP [Cd(2-tpc)_2_(4-bphz)_2_]_n_ (2-tpc=2-thiophenecarboxylate) [26] where only one of the azine ligands works as a bidentate linker, while the second one is terminal and coordinates in a monodentate mode.

**Figure 3:**
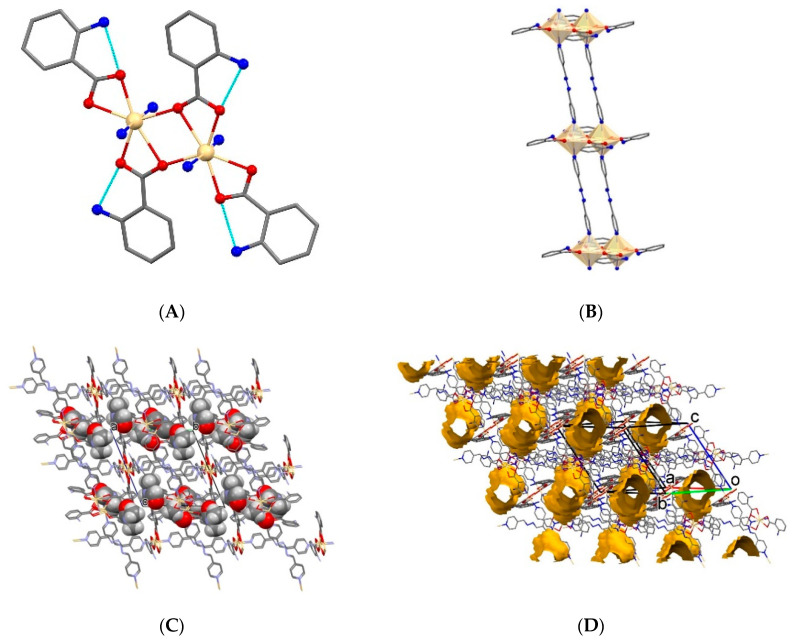
(**A**) Coordination surrounding of metals illustrating the secondary building units (SBU) in **2**. (**B**) Fragment of ladder-type coordination chain in **2**. (**C**) Crystal packing with the dmf solvent. (**D**) The SAVs in **2** are shown in yellow. H-atoms are omitted.

Compound **2** also crystallizes in the form of a ladder-type coordination chain, but with the binuclear [Cd_2_(CO_2_)_4_] secondary building units (SBU) as ladder steps. Such SBU is often observed in Cd coordination compounds with carboxylate ligands. The asymmetric unit comprises two Cd(II) atoms, four 2-aba residues, and two 4-bphz ligands as components of two chemically identical but crystallographically unique ladder-type coordination chains. Each Cd(II) atom takes an N_2_O_5_ pentagonal-bipyramidal geometry with five O atoms from three aba residues situated in the basal plane and two N-atoms from the azine ligands situated in *trans*-axial positions (Figure 3A). Two neighbouring metal polyhedra related to the inversion center share the common O···O edge in the basal plane and result in the H-shaped four-connected [Cd_2_(CO_2_)_4_] SBU (Figure 3B). One aba residue coordinates in the bidentate-chelate (µ_1_-η^2^) mode, two other-in the bidentate-bridging and chelate (µ_2_-η^2^:η^1^) mode providing the Cd···Cd separations in the centrosymmetric binuclear units of 3.8890(9) Å and 3.8959(9) Å in two chains. The close proximity of extension points of SBU together with bulky pyridine moiety of 4-bphz ligands provides the parallel arrangement of these ligands in a polymer chain. The Cd···Cd separations across the bridging 4-bphz chromophores are equal to 15.884(1) Å and 15.858(1) Å in two chains. The double chains stack in the crystal in the criss-cross packing mode (Figure 3C). The architecture of the coordination chains bears a resemblance to the one mentioned above 1D CPs with 2-thiophenecarboxylate [26]. Identically, the criss-cross crystal packing in **2** is governed by the accommodated dmf molecules as templates [11]. The criss-cross packing of coordination chains results in the channel voids decorated with phenyl rings of the aba residues and occupied by the dmf guest molecules. The guest-accessible channels run along the [−110] crystallographic direction and comprise 18.6% or 542.6 Å^3^ of the unit cell volume (Figure 3C,D).

#### 2.2.2. 2D Coordination Polymers

The extension of crystal structures to 2D coordination polymers was achieved by replacing the monocarboxylic 2-aminobenzoic acid with the dicarboxylic acids namely, aliphatic conformationally flexible sebacic acid, aromatic semirigid homopthalic acid, or rigid isophthalic acid, which resulted in five compounds [Cd(seb)(4-bphz)]_n_·n(H_2_O) (**3**), [Cd(seb)(4-bpmhz)]_n_·n(H_2_O) (**4**), [Cd(hpa)(3-bphz)]_n_ (**5**), [Zn(1,3-bdc)(3-bpmhz)]_n_·n(MeOH) (**6**), and [Cd(1,3-bdc)(3-bpmhz)]_n_·0.5n(H_2_O)·0.5n(EtOH) (**7**), increasing the connectivity of [Cd_2_(CO_2_)_4_] SBU to eight. The double covalent cross-linking of such di-nuclear units in **3**–**7** results in similar 2D coordination frameworks with 4,4-topology and different solvents situated in the interlayer space. Only compound **5** is solvent-free.

**Figure 4:**
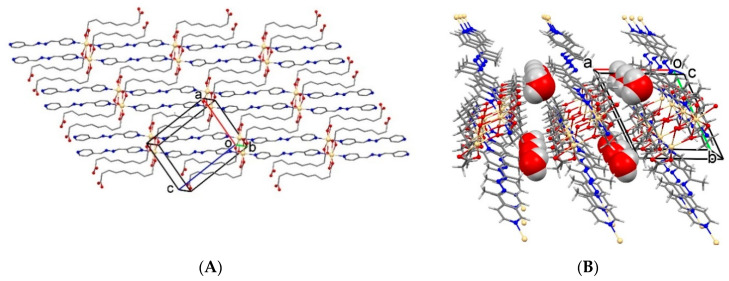
(**A**) Fragment of the coordination layer parallel to the (ac) plane in **3**. C-bound H-atoms are omitted. (**B**) Side view of three coordination layers with accommodated water molecules shown in space-filling mode in **4**.

**Figure 5:**
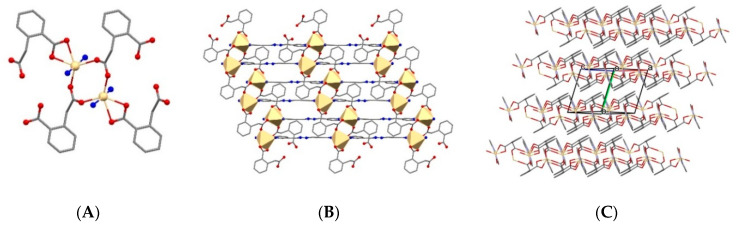
(**A**) Coordination surrounding of metals illustrating the SBU in **5**. (**B**) Top view of the coordination layer. (**C**) Packing of coordination layers. Side view. H-atoms are omitted.

**Figure 6:**
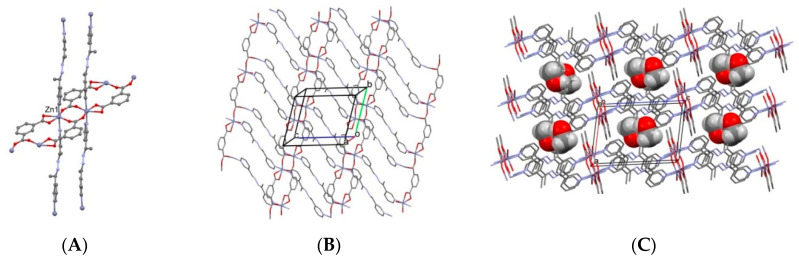
(**A**) Fragment of the coordination layer in **6**. (**B**) Top view of the coordination layer. (**C**) Side view of three interdigitated coordination layers with accommodated MeOH molecules shown in a space-filling mode. Solvent molecule H-atoms are omitted.

**Figure 7:**
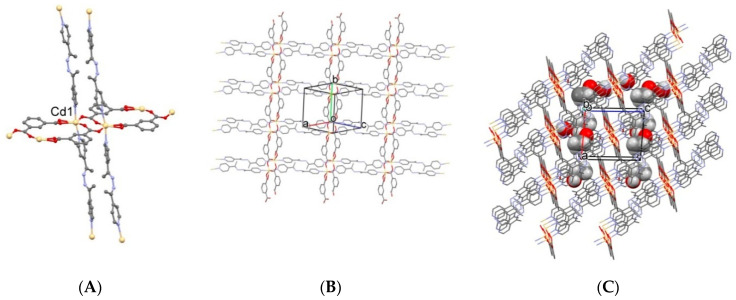
(**A**) Fragment of the coordination layer in **7**. (**B**) Top view of the coordination layer. (**C**) Packing of the interdigitated layers with accommodated EtOH/H_2_O molecules shown in a space-filling mode. Solvent molecule H-atoms are omitted.

The asymmetric units in **3**–**7** comprise one metal cation, one dicarboxylate anion, and one azine ligand. The fragments of coordination frameworks and crystal packings for **3**–**7** are shown in Figure 4, Figure 5, Figure 6 and Figure 7. In **3** and **4** that differ only by the methyl groups in the azine linkers, the Cd atom similar to **2**, has an N_2_O_5_ pentagonal bipyramidal coordination core coordinated by three carboxylate residues in the basal plane and two azine ligands in axial positions (Figure 4A, Table S1 in Supplementary Materials). For compounds **5**–**7**, although the metal ions contact with the same number of carboxylate residues, the number of metal-O coordination bonds is reduced due to a bidentate-bridging (µ_2_-η^1^:η^1^) coordination mode of two carboxylic groups in **5**–**7** instead of bidentate-bridging and the chelate (µ_2_-η^2^:η^1^) mode in **2**–**4**. That resulted in the N_2_O_4_ distorted octahedral coordination geometries of metal. In the binuclear [Cd_2_(CO_2_)_4_] unit, the metal polyhedra have no common edge (Figure 5A, Figure 6A, and Figure 7A, Table S1). Likely, conformation flexibility of the seb residue favors more effective involvement of carboxylic group in coordination in **3** and **4**. The different modes of coordination of two carboxylic groups in **3**–**7**, chelate, and bidentate-bridging, were also established by careful examination of asymmetric and symmetric stretching frequencies of carboxylate groups in Fourier transform infrared (FTIR) spectra.

The coordination layers in **3**–**7** are built similarly, from ladder-type coordination chains analogous to those observed in **2**, which are double cross-linked in the coordination layers by dicarboxylate ligands (Figure 4A, Figure 5B, Figure 6B, Figure 7B). In **3** and **4**, the chains are double linked by seb ligands with the shortest metal...metal separation across this ligand of 11.664 Å in **3** and 12.592 Å in **4**. The metal-metal separations in the binuclear units are 3.8379(6) Å and 3.8560(4) Å for **3** and **4**, respectively. The Cd···Cd separations in the binuclear units **5**, **7** are equal to 3.9706(4) and 4.1550(5) Å, respectively, and 4.1537(5) Å for Zn-based network in **6**. The metal-metal separations across the azine ligands with different N-positions vary in the ranges of 15.761(1)–15.986(1) Å and 14.312(11)–14.579(1) Å for straight and bent-coordinating azine ligands, respectively. The azine ligands in **3**–**6** are in virtually planar conformations and situated in the virtually parallel planes.

The reported sources indicated the significant solvent and gas uptakes by the alike systems [22,23,24]. Therefore, the specificity of packing of coordination layers and inclusion of solvents is worth studying for the reported 2D networks. Compounds **3**–**7** were obtained from the protic solvents, either water or water-alcohol mixtures, and demonstrate different solvent uptakes. Compounds **3** and **4** both crystallize as monohydrates. The layers stack along the crystallographic *b* axis in **3** (Figure 4B) and *a* axis in **4** (Figure 5C). Compound **3** reveals the disordering of one seb anion synchronized with the weak attachment of the water molecule to the carboxylate backbone via one OH···O H-bond, O(1W)-H(1W)···O(2) (1.95(3), 2.817(7) Å, ∠OHO 169(10)°). Otherwise, in the perfectly ordered structure **4** where the water molecule acts as a double H-donor and a single H-acceptor, it glues the coordination layers via three hydrogen bonds, O(1W)-H(1C)···O(2)(x + 1, y, z + 1) (2.03, 2.874(4) Å, ∠OHO 169.2°), O(1W)-H(1D)···O(1)(1 − x, −y, 1 − z) (2.06, 2.912(4) Å, ∠OHO 175.6°), C(4)-H(4) ···O(1W) (2.32, 3.177(4) Å, ∠OHO 152.7°). In **3, 4**, both ligands are situated in the layers′ planes, and the layers do not interdigitate. In the solvent-free compound **5**, the coordination layers stack along the crystallographic *b* axis (Figure 6c), and meet by the walls of the hpa residues that are situated parallel to the layer′s mean plane thanks to the methylene bridge in the hpa moiety. The layers also do not interdigitate and are held together by van der Waals interactions. In **6** and **7**, the layers pack in the interdigitation mode due to the ipa perpendicular arrangement to the layers′ mean planes with the compartments between the parallel double walls of ipa-residues filled by the disordered solvents. The replacement of 3-bpmhz ligand used in **6** by its positional isomer 4-bpmhz in **7** is accompanied by the increase in metal…metal separation in one direction in the 2D coordination network, and gives rise to the rectangular windows in the coordination network (Figure 7B). Thus, the straight or bent-coordinating to the metals azine ligands together with different flexibility dicarboxylic partners provide access to more or less dense coordination layers.

**Figure 8:**
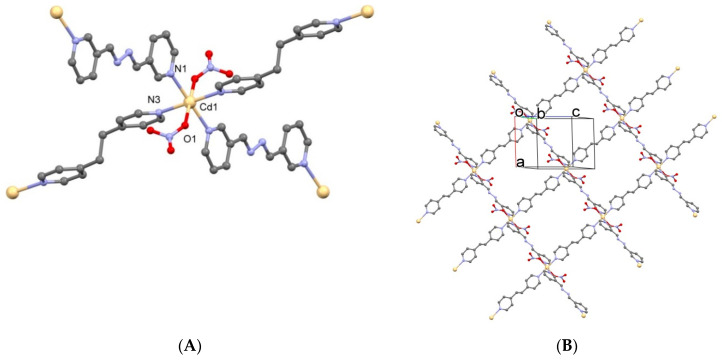

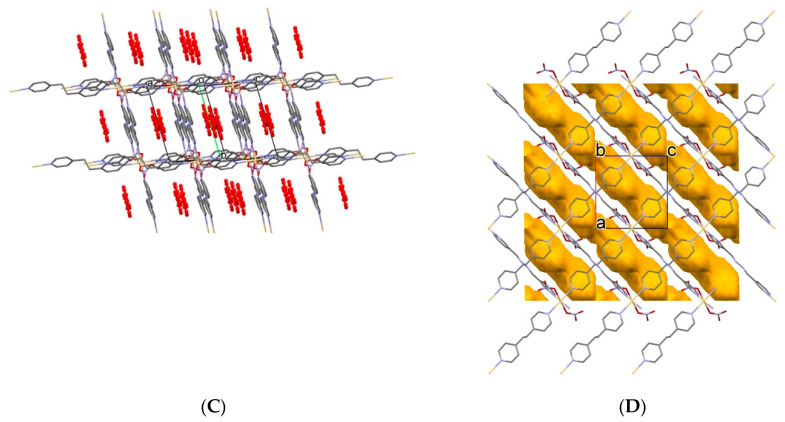
(**A**) Coordination environment of Cd(II) atom in **8**. (**B**) Top view of the coordination layer. (**C**) Packing of coordination layers with accommodated 3-bphz molecules colored red. (**D**) Guest-accessible channels in **8** shown in yellow. H-atoms are omitted.

Similar to **1**, the 2D coordination polymer [Cd(NO_3_)_2_(3-bphz)(bpe)]_n_·n(3-bphz) (**8**) possesses the Cd(II) atom as a node. The asymmetric unit comprises one half of the Cd(II) atom, one half of the bpe ligand, and two halves of the 3-bphz ligand, coordinated and non-coordinated ones, together with one nitrate anion. The Cd(II) atom resides on an inversion center and takes a distorted octahedral geometry with the N_4_O_2_ set of donor atoms going from two bpe, two 4-bphz ligands, and two nitrato-groups, identical ligands being situated in trans-positions (Figure 8A). The Nitrato-group acts in a monodentate mode. Two neutral ligands act in the bidentate-bridging modes and provide the linking of the components in the layer of (4,4′) topology [27] parallel to the (121) crystallographic plane (Figure 8B). Within the layer, the Cd···Cd separations are of 13.901 and 14.623 Å. The stack of parallel layers forms the channels filled by the 3-bphz guest molecules that are organized in the virtually flat chains via a couple of weak intermolecular interactions, C(16)-H(16)···N(5)(2 − x, 1 − y, 2 − z) 2.65, 3.424(4) Å; ∠CHN 140.89° in the form of an R_2_^2^(6) supramolecular synthon. The chains of guest molecules pack roughly parallel (8.7°) to the walls formed by the coordinated bpe ligands (Figure 8C) and form infinite stacking columns with the alternating Cg···Cg separations of 4.997 and 5.405 Å. The rectangular channels that run in the crystal along the [−101] direction constitute 25.4% or 232.02 Å^3^ of the unit cell volume (Figure 8d).

### 2.3. IR Spectroscopic Characterization

The FTIR (attenuated total reflectance=ATR) spectra for **1**–**8** are summarized in Figure S1. The bands ν_as_(COO^−^) and ν_s_(COO^−^) in the spectra **1**–**7** indicate the presence of carboxylate groups coordinated to the metal. The IR spectra for compounds **1** and **2** have similar profiles. For both compounds, the band ν_as_(COO^−^) is registered at 1511 cm^−1^ and the band ν_s_(COO^−^) is registered at 1389 cm^−1^ for **1** and at 1381 cm^−1^ for **2**. At the same time for **1**, the bands of low intensity at 1417 and 1303 cm^−1^ can be assigned to the ν_as_(NO) and ν_s_(NO) vibrations of the nitrato-group. The DMF solvent is identified by the strong ν(C=O) band at 1669 cm^−1^ for **2** and less intensive band at 1663 cm^−1^ for **1**. The presence of the amino group is confirmed by the ν_as_(NH) bands at 3445 cm^−1^ in both compounds and ν_s_(NH) at 3314 cm^−1^ for **1** and 3320 cm^−1^ for **2**. In compounds **3** and **4**, the sebacate anion was identified by two strong bands at 1541 cm^−1^ and 1416 cm^−1^. The homophthalate anion in **5** and isophthalate anions in **6** and **7** were registered by the pairs of bands at 1551 and 1387 cm^−1^ for **5**, 1549, and 1390 cm^−1^ for **6**, and 1542, and 1382 cm^−1^ for **7**. The nitrate anion in **8** was registered by the strong bands ν_as_(NO) and ν_s_(NO) at 1419 and 1301 cm^−1^, respectively. The characteristic band of the azomethine group ν(C=N) was identified in the range ~1630–1610 cm^−1^. The H_2_O crystallization molecules in compounds **3**, **4**, and **7** can be identified by the bands ν(OH) in the region ~3350 cm^−1^ and δ(H_2_O) at 1662 cm^−1^ for **4**.

### 2.4. Desolvation, Solvent-Exchange, and Adsorption Properties

**Figure 9:**
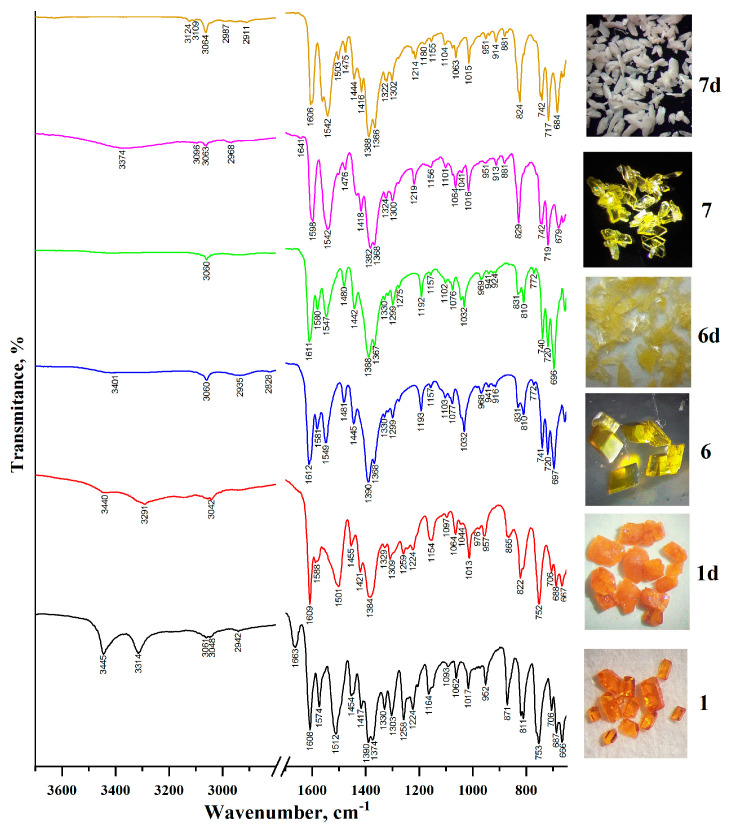
IR spectra and crystal images of as-synthesized and de-solvated forms **1**/**1d**, **6**/**6d**, and **7**/**7d**, respectively.

**Figure 10:**
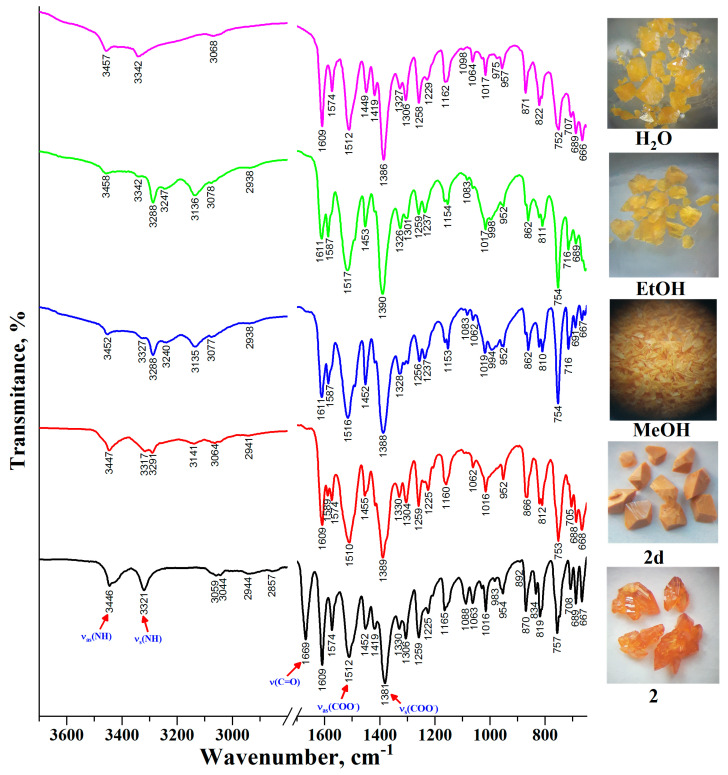
IR spectra and crystal images of compound **2**/**2d**, and solvent-exchange products.

The de-solvated forms **1d**, **2d**, **6d**, and **7d** were obtained by the heating of as-synthesized compounds **1**, **2**, **6**, and **7** at specific temperatures on the glycerine bath in vacuo for 10 h. Figure 9; Figure 10 show the images of the as-synthesized and de-solvated crystals and their IR spectra that confirm the crystals integrity and elimination of solvated molecules. Compound **1** was de-solvated at 170 °C. The crystals **1d** have kept their shape but lost the luster, and the orange color intensified. The lack of the dmf molecules is indicated by the missing of the ν(C=O) band at 1663 cm^−1^ (Figure 9). Compound **2** was de-solvated at 150 °C. The crystals kept their shape and crystallinity intact (Figure 10, Figure S2). The lack of the DMF molecules is indicated by the missing of the ν(C=O) band at 1669 cm^−1^ (Figure 10). The crystals of compound **6** de-solvated at 100 °C decomposed into thin, transparent plates. In the case of compound **7**, the crystals heated at 120 °C turned white, and revealed the deterioration of the crystal quality while retaining its crystallinity (Figure S3). This is in line with some reported examples for the relative systems [22]. However, we have not yet succeeded in determining the crystal structure of the de-solvated forms of these frameworks.

For the activated forms **2d** and **7d**, the N_2_ adsorption isotherms were registered at 77 K. The N_2_ sorption isotherms display a typical V-type shape and indicate negligible gas uptake (Figure S4) most likely due to kinetic and temperature effects and weak N_2_ interactions with the framework′s walls [21].

The favorable criss-cross packing of double chains in compound **2** led to the formation of channels where the dmf guest molecules are located. Like 1D CP [Cd(4-bpmhz)(2-tpc)_2_]_n_^.^0.5n(4-bpmhz) that revealed solvent-exchange properties [26], similarly organised **2** was studied for possible solvent exchange. The crystals **2** were immersed into solvents MeOH, EtOH, and H_2_O. The changes in the compound were examined by IR spectroscopy and X-ray powder diffraction (XRPD) analysis (Figure 10 and Figure S2). Upon sucking the crystals into MeOH, after a short time, their destruction took place with the formation of an orange polycrystalline product (Figure 10). After holding in solution for 5 min, this product was isolated, and the recorded IR spectrum clearly indicated that the DMF molecules were substituted with MeOH molecules. The ν(C=O) band disappeared from the spectrum, and the MeOH molecules can be highlighted by the broad band ν(OH) in the region ~3500–3000 cm^−1^ and the band ν(C-O) at 994 cm^−1^ (Figure 10). The shifts of the bands for the amino and carboxylato-groups were also registered, likely due to the formation of hydrogen bonds with the solvent molecules. The same observations, which substitute DMF molecules, were registered in the case of EtOH and H_2_O solvents. However, the exchange of solvents proceeds more slowly and the crystals are more stable. After 1 h of keeping the crystals in solution, the IR spectra were recorded (Figure 10). The comparison of XRPD patterns reveals that compound **2** retains its crystallinity (Figure S2). The same experiments were performed with the crystals of compound **1**, which also contain DMF solvent molecules. Surprisingly, they proved to be quite stable in these solvents. In the EtOH solution after a month, the crystals retain perfectly their shape and transparency and the recorded IR spectra do not attest to the exchange of DMF molecules.

### 2.5. Photoluminescence Properties

**Figure 11:**
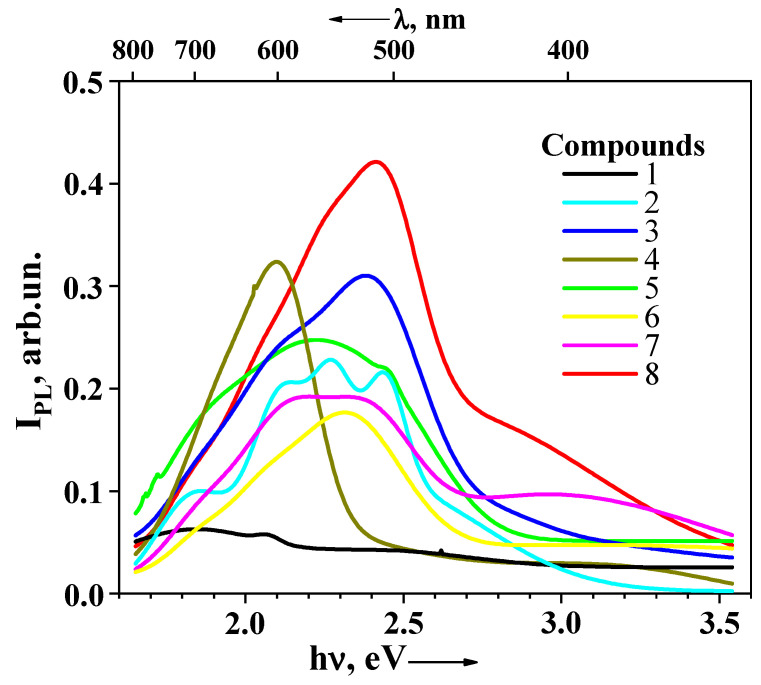
Solid-state emission plots for compounds **1**–**8**.

The possibility for tuning the emission properties of Zn(II) and Cd(II) mixed-ligand coordination networks obtained via interplay of N-pyridine type ligands and dicarboxylic acids has been shown [28]. Otherwise, the ligand-based emission properties have been registered for the coordination networks with the azine chromophores used in these studies [26,29,30,31,32,33,34,35,36,37,38,39]. In continuation of those studies, herein, the emission properties of as-synthesized coordination polymers **1**–**8**, and de-solvated compounds **1d**, **2d**, **6d**, and **7d** are reported. The photoluminescence (PL) spectra were excited by a pulsed nitrogen laser (λ_exc_ = 337.1 nm) at room temperature and recorded in the visible region of the spectrum (Figure 11). The various frameworks showed unique emission spectra. The emission spectra are dominated by the output from their azine ligands [26] with peaks centered between 650–390 nm. For the bands′ deconvolution, the Gaussian function was used, which allowed us to resolve the spectra as superpositions of several (at least five for almost all samples) radiative processes with maxima registered at 1.9 eV (650 nm), 2.08 eV (595 nm), 2.2 eV (563 nm), 2.45 eV (515 nm), 2.8 eV (442 nm), and 3.15 eV (390 nm), whose intensities vary from sample to sample. The highest enhancement in fluorescence was observed in the case of compound **8** with the dominant peak at 2.45 eV (515 nm). The significant emission attenuation for **1**–**7** originates from the presence of protic solvents (H_2_O, MeOH, EtOH) as emission quenchers [40] in the crystal pores with almost complete emission decay registered for porous **1** (Figure 11). The meaningful emission for **8** can be explained by the lack of quenching solvents. The favorable arrangement of the confined 3-bphz chromophore as a part of the infinite chromophores′ stacking columns (Figure 8c), and the double amount of chromophore in **8** compared with **1**–**7**. The dominant intensity of the peak at 2.45 eV (515 nm) decreases in a row **8**→**6**→**3** →**2**→**7**→**5**. The peak at 2.08 eV (595 nm) likely is determined by the impact of double chromophore pillars being the fragments of coordination arrays in **2**–**7** (Figure 3a, Figure 4a, Figure 5a, Figure 6a, Figure 7a, and Figure 8a) with the overlay of their pyridine rings with the Cg···Cg separations ca. 3.6 Å as imitating the emitting H-aggregates [41]. The significance of the peak at 2.08 eV (595 nm) increases in a row **8**→**5**→**3**→**2**→**7**→**6**→**4** in the emission spectra. A significant contribution to the formation of the PL spectra in isostructural compounds **3** and **4** is made by the long-wavelength peaks. The red-shift for **4** compared with **3** is explained by the extension of the planar backbone of the 4-bpmhz chromophore compared with the non-methylated analogue, accompanied by the previously mentioned rigidification of the crystal lattice through the tightly bound water molecules and the lack of disordering [42]. The profile of the PL spectrum for **2** with the well-resolved peaks at 1.9 (650 nm), 2.08 (595 nm), 2.2 (563 nm), and 2.45 eV (515 nm) is most likely connected with the specificity of crystal packing in this compound that includes overlapping pyridine rings and also close azine-bridges and their participation in the infinite stacking (Figure 3b). The short-wavelength luminescence band at 400 nm (manifested as a shoulder) does not contribute to emission profile in all samples. The contribution decreases in a row **7**→**4**→**8**→**6**→**5**→**3**→**2**.

**Figure 12:**
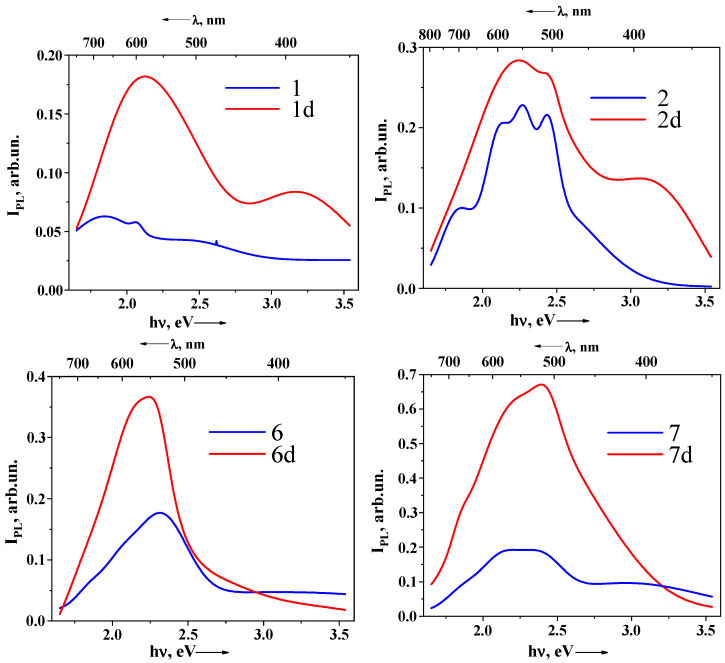
Comparable solid-state emission plots for as-synthesized **1**, **2**, **6**, and **7** and de-solvated **1d**, **2d**, **6d**, and **7d** compounds.

When we move from as-synthesized compounds to their de-solvated forms, we observe the retention of shapes of the PL plots in the pairs **1**/**1d**, **2**/**2d**, and **6**/**6d** and redistribution of emission peaks for pair **7**/**7d** accompanied by the significant increase in intensity of emission for the de-solvated forms (Figure 12), which allow suggesting these systems as sensors for small molecules.

**Figure 13:**
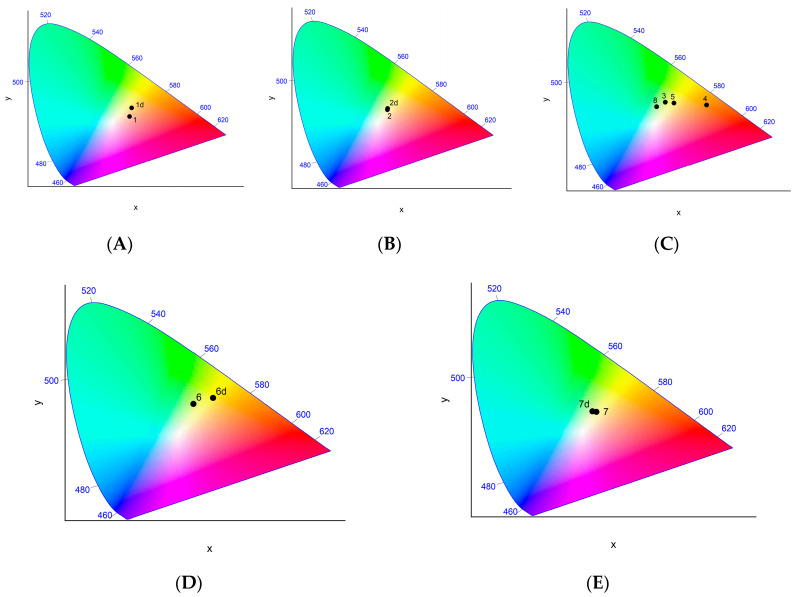
(**A**) CIE (Commission Internationale de l′Éclairage) diagram for the emission spectra of **1**/**1d**, (**B**) **2**/**2d**, (**C**) **3**–**5**, **8**, (**D**) **6**/**6d**, and (**E**) **7**/**7**d showing tunability of emission.

The emission spectra were translated into chromaticity coordinates and plotted onto CIE (Commission Internationale de l′Éclairage) diagrams that allow visualizing the tunability of the emissions and indicate that the majority of compounds reveal the chromaticity coordinates close to the desirable human-friendly white-yellow region (Figure 13). Due to similarities in the crystal structures, the CIE coordinates are close to each other for **3** (0.35213, 0.43764), **4** (0.49977, 0.42363), **5** (0.38311, 0.43358), **6** (0.355, 0.44617), **7** (0.35193, 0.4048), and **8** (0.32094, 0.41541). The CIE coordinates for compounds **1** (0.37693, 0.35899) and **2** (0.3482, 0.39261) show the values closest to those of pure white light (0.33, 0.33). Since the main intensity of emission for all samples is concentrated in the range 500–600 nm, they may be considered as useful materials for making physiologically-friendly yellow and yellow-green lighting sources.

## 3. Materials and Methods

Zinc and cadmium nitrates, 4-pyridinecarboxaldehyde, 3-pyridinecarboxaldehyde, 4-acetylpyridine, 3-acetylpyridine, hydrazine sulfate, 2-aminobenzoic acid, sebacic acid, homophthalic acid, benzene-1,3-dicarboxylic acid, 1,2-bis(4-pyridyl)ethane, and solvents were obtained from commercial sources (Sigma-Aldrich, St. Louis, MO, USA) and were used without further purification. The IR (ATR) spectra were recorded on an FTIR Spectrum-100 Perkin Elmer spectrometer in the range of 650–4000 cm^−1^. Elemental analysis was performed on a vario EL III Element Analyzer. Solid state emission spectra were recorded using a pulse nitrogen laser (λ_exc_ = 337.1 nm) at 300 K. The excitation pulse duration was 15 ns, the repetition frequency was 50 Hz, and the pulse energy was 0.2 mJ. The emission was detected with an FEU-79 instrument (multialkaline photocathode Sb (Na_2_K) with the adsorbed cesium layer on the surface, characteristic of the S20 type). The intrinsic time of the detecting system was 20 ns. The afterglow duration (at 300 K) for all the studied compounds was shorter than the time resolution of the detecting system. Gas adsorption parameters were obtained from N_2_ adsorption isotherms at 77 K. The adsorption isotherms were measured using Autosorb-1-MP (Quantachrome). The specific surface area (SBET) was calculated using the Brunauer−Emmett−Teller (BET) equation. The total pore volume (*V*t) was calculated by converting the amount of N_2_ gas adsorbed at a relative pressure of 0.99 to equivalent liquid volume of the adsorbate (N_2_). The guest evacuation from compounds was performed in the glycerine bath under a vacuum created by the water jet vacuum pump, maintaining a constant temperature. X-ray powder diffraction data were collected with a DRON-UMB X-ray powder diffractometer equipped with an Fe-Kα radiation (λ = 1.93604 Å) source. The diffractometer was operated at 30 kV and 30 mA. The data were collected over an angle range of 5°–50° at a scanning speed of 5° per minute.

## 4. X-ray Crystallography

Single crystal X-ray diffraction measurements for **1**–**8** were carried out on an Xcalibur E diffractometer equipped with a CCD area detector and a graphite monochromator utilizing MoKα radiation at room temperature. Final unit cell dimensions were obtained and refined on entire data sets. All the calculations to solve the structures and to refine the models proposed were carried out with the programs SHELXS97 and SHELXL2014 [43,44]. Hydrogen atoms attached to carbon atoms were positioned geometrically and treated as riding atoms using SHELXL default parameters with U_iso_(H) = 1.2 U_eq_(C) and U_iso_(H) = 1.5 U_eq_(CH_3_). Whenever necessary, restraints were imposed on geometry and displacement parameters of disordered molecules. The disordering problems were resolved in **1**–**3**, and **6**–**8**, which include the disordered fragments in the coordination frameworks: one aba anion in **2**, one seb anion in **3**, methyl groups of 3-bpmhz ligand in **6**, 4-bpmhz ligand in **7**, and one nitrato-anion in **8**, and the solvent molecules that occupy the pores in the crystal lattices. The disordered fragments of coordination networks over two positions were refined, keeping the total single occupancy. The outer-sphere molecules were modeled as solvents used in the syntheses in **1**–**7**, and the confined 3-bphz chromophore in **8**. The disordered solvents were refined with overall single occupancies in **1**, **3**, **4**, **6**, and **7**, and with 1.5 occupancies for two disordered dmf molecules in **2**. Crystals of all reported compounds are triclinic and the X–ray data and the details of the refinement for **1**–**8** are summarized in Table S2. Compounds **1**, **3**–**8** each comprise one formula unit in the asymmetric unit (Z′ = 1) while compound **2** comprises two formula units (Z′ = 2) in the asymmetric unit. The principle bond distances and angles in **1**–**8** have the common values and are summarised in Table S1. The figures were produced using MERCURY [45]. The solvent accessible areas were evaluated using MERCURY [45] and PLATON [46] facilities. Further details of the crystal structure investigation for compounds **1**–**8** may be obtained free of charge from the Cambridge Crystallographic Data Centre (CCDC) (https://www.ccdc.cam.ac.uk/structures/) by quoting the CCDC deposition numbers 2022947–2022954.

## 5. Synthetic Procedures

### Synthesis of the Schiff-Base Ligands

In the reported source [47], the hydrazine hydrate was used in the synthesis of these ligands. We used the hydrazine sulfate salt by adding sodium carbonate to bring the solution to a neutral pH. The synthetic protocols and spectral details are given in References [29,30].

*[Cd(2-aba)(NO_3_)(4-bphz)_3/2_]_n_·n(dmf)* (**1**), Cd(NO_3_)_2_·4H_2_O (0.03 g, 0.01 mmol), 2-aminobenzoic acid (0.013 g, 0.1 mmol), and 4-bphz (0.042 g, 0.2 mmol) were dissolved in 3 mL of DMF. The solution was filtered and allowed to evaporate slowly at room temperature. After five days in the solution, orange-red crystals form. Yield: ~34%. Anal. Calc. for C_25_H_20_CdN_8_O_5_(dmf), (%): C, 48.18; H, 3.90; N, 18.06; O, 13.75. Found: C, 48.15; H, 3.08; N, 17.99; O, 13.24. IR-ATR (cm^−1^): 3445 m, 3314 m, 3061 w, 2941 w, 1663 m, 1608 s, 1573 s, 1511 s, 1454 m, 1417 s, 1389 s, 1373 s, 1329 s, 1303 s, 1258 s, 1223 s, 1205 m, 1164 m, 1062 m, 1016 m, 952 m, 871 s, 819 s, 811 s, 753 s, 705 s, 687 s, 666 s.

*[Cd(2-aba)2(4-bphz)]n·0.75n(dmf)* (**2**), additionally, in 5 mL DMF were dissolved on heating Cd(NO_3_)_2_·4H_2_O (0.03 g, 0.01 mmol), 2-aminobenzoic acid (0.027 g, 0.2 mmol), and 4-bphz (0.042 g, 0.2 mmol). Furthermore, 1 mL of Et_3_N solution (0.2 M) dissolved in DMF was added to this solution. The solution was allowed to evaporate slowly at room temperature. After four days, yellow-orange crystals form in the solution. Yield: ~74%. Anal. Calc. for C_26_H_22_CdN_6_O_4_(0.75dmf), (%): C, 52.22; H, 4.23; N, 14.56; O, 11.71. Found: C, 52.15; H, 3.88; N, 14.39; O, 11.53. IR-ATR (cm^−1^): 3445 m, 3320 m, 3058 w, 3043 w, 2943 w, 2856 w, 1669 s, 1609 vs, 1573 s, 1511 s, 1452 s, 1418 s, 1381 vs, 1329 s, 1305 s, 1258 s, 1224 m, 1164 m, 1087 m, 1063 m, 1015 m, 953 m, 870 m, 834 m, 818 m, 756 s, 708 m, 689 m, 667 m.

*[Cd(seb)(4-bphz)]_n_·n(H_2_O)* (**3**), Cd(NO_3_)_2_·4H_2_O (0.03 g, 0.1 mmol) was dissolved in 6 mL of H_2_O. This solution was added to a test tube. Therefore, 2 mL of H_2_O: EtOH solution (1:1) was carefully added to this solution. Thereafter, the solution was composed of 4-bphz (0.03 g, 0.14 mmol), sebacic acid (0.02 g, 0.1 mmol), and a drop of NaOH (2 M solution), which was dissolved in 6 mL of EtOH that was added. The tube was covered with parafilm and left at room temperature. After 5 days, in the middle of the tube, yellow needle-shaped crystals formed. Yield: ~62%. Anal. Calc. for C_22_H_26_CdN_4_O_4_(H_2_O), (%): C, 48.85; H, 5.22; N, 10.36; O, 14.79. Found: C, 48.81; H, 5.15; N, 10.28; O, 14.70. IR-ATR (cm^−1^): 3654 w, 3361 m, 3041 w, 2957 m, 2921 s, 2853 m, 1640 w, 1629 w, 1607 s, 1541 vs, 1468 m, 1416 vs, 1366 m, 1304 s, 1238 m, 1222 m, 1175 m, 1120 m, 1090 w, 1063 m, 1014 s, 972 m, 955 m, 903w, 887 w, 873 w, 823 s, 730 w, 687 s.

*[Cd(seb)(4-bpmhz)]n·n(H2O)* (**4**), the compound was obtained under the same conditions as **3**. After five days, in the middle of the tube, orange crystals formed. Yield: ~57%. Anal. Calc. for C_24_H_30_CdN_4_O_4_(H_2_O), (%): C, 50.67; H, 5.67; N, 9.85; O, 14.06. Found: C, 50.58; H, 5.61; N, 9.82; O, 13.98. IR-ATR (cm^−1^): 3343 br. m, 3080 w, 3019 w, 2922 m, 2843 m, 1662 w, 1608 s, 1542 vs, 1495 m, 1462 m, 1447 m, 1416 vs, 1368 s, 1325 m, 1311 s, 1290 s, 1262 m, 1241 m, 1219 m, 1177 w, 1124 m, 1063 s, 1015 s, 973 w, 901 m, 824 s, 792 s, 759 s, 723 s, 679 s, 665 s.

*[Cd(hpa)(3-bphz)]_n_* (**5**), the compound was obtained under the same conditions as **3**. After one week, in the middle of the tube, yellow crystals formed. Yield: ~55%. Anal. Calc. for C_21_H_16_CdN_4_O_4_, (%): C, 50.37; H, 3.22; N, 11.19; O, 12.78. Found: C, 50.25; H, 3.10; N, 11.03; O, 12.66. IR-ATR (cm^−1^): 3110 w, 3075 w, 3036 w, 2969 w, 2891 w, 1632 m, 1594 vs, 1578 vs, 1551 vs, 1486 m, 1448 m, 1423 s, 1387 vs, 1305 s, 1285 m, 1276 m, 1251 m, 1230 m, 1187 s, 1154 m, 1098 m, 1054 m, 1029 s, 972 m, 858 s, 833 s, 806 s, 761 s, 734 vs, 696 vs, 671 vs.

*[Zn(1,3-bdc)(3-bpmhz)]n·n(MeOH)* (**6**), Zn(NO_3_)_2_·6H_2_O (0.03 g, 0.1 mmol) and isophthalic acid (0.016 g, 0.1 mmol) were dissolved in 6 mL MeOH. The 3-bpmhz solution (0.05 g, 0.2 mmol) dissolved in 6 mL of MeOH was added to this solution. The glass was covered with parafilm. After three days, yellow crystals form in the solution. Yield: ~47%. Anal. Calc. for C_22_H_18_N_4_O_4_Zn(MeOH), (%): C, 55.27; H, 4.44; N, 11.21; O, 16.00. Found: C, 55.16; H, 4.37; N, 11.15; O, 15.98. IR-ATR (cm^−1^): 3425 br. w, 3059 w, 2935 w, 1612 vs, 1581 s, 1548 vs, 1481 m, 1444 s, 1390 vs, 1367 vs, 1329 m, 1299 s, 1275 m, 1192 s, 1156 w, 1103 m, 1076 m, 1032 s, 968 m, 940 w, 915 w, 830 s, 810 s, 772 w, 740 vs, 720 vs, 696 vs, 655 s.

*[Cd(1,3-bdc)(4-bpmhz)]_n_·0.5(H_2_O)·0.5(EtOH)* (**7**), Cd(NO_3_)_2_·4H_2_O (0.03 g, 0.1 mmol) and isophthalic acid (0.016 g, 0.1 mmol) were dissolved in 12 mL of EtOH and 6 mL of H_2_O. The 4-bpmhz solution (0.05 g, 0.2 mmol) dissolved in 12 mL of EtOH was added to this solution. The glass was covered with parafilm. After one week, yellow crystals form in the solution. Yield: ~51%. Anal. Calc. for C_22_H_18_CdN_4_O_4_(0.5H_2_O·0.5EtOH), (%): C, 50.52; H, 4.05; N, 10.25; O, 14.63. Found: C, 50.39; H, 3.95; N, 10.18; O, 14.61. IR-ATR (cm^−1^): 3374 br. w, 3063 w, 2967 w, 1644 w, 198 vs, 1541 vs, 1501 m, 1476 m, 1435 s, 1417 s, 1382 vs, 1368 vs, 1323 s, 1300 s, 1219 s, 1156 m, 1101 m, 1064 s, 1016 s, 951 m, 912 m, 881 m, 828 vs, 747 vs, 719 vs, 678 vs, 663 vs.

*[Cd(NO_3_)_2_(3-bphz)(bpe)]_n_·n(3-bphz)* (**8**), Cd(NO_3_)_2_·4H_2_O (0.03 g, 0.1 mmol), bpe (0.018 g, 0.1 mmol), and 3-bphz (0.021 g, 0.1 mmol) were dissolved in 6 mL of DMF. The mixture was stirred on heating for 5 min. Then the solution was filtered off and allowed to evaporate slowly. After a week, pale yellow crystals form in the solution. Yield: ~56%. Anal. Calc. for C_24_H_22_CdN_8_O_6_(3-bphz), (%): C, 51.41; H, 3.83; N, 19.98; O, 11.41. Found: C, 51.38; H, 3.75; N, 19.84; O, 11.36. IR-ATR (cm^−1^): 3039 w, 2935 w, 1628 s, 1609 s, 1585 m, 1560 w, 1482 m, 1419 vs, 1301 vs, 1228 s, 1189 s, 1118 m, 1095 m, 1037 s, 1013 s, 975 s, 948 s, 872 m, 862 m, 824 vs, 806 vs, 708 s, 697 vs.

## 6. Conclusions

In summary, the strategy of replacing monocarboxylic acids (2-aminobenzoic) by three dicarboxylic acids (sebacic, homophthalic, and isophthalic) with different skeleton flexibilities in a blend with N, N′-diazine ligands has succeeded for extending the dimensionality of coordination polymers from 1D to 2D while keeping invariable the coordination polymeric chains originated from azine ligands. Seven new azine/carboxylate coordination networks were obtained and characterized, namely, [Cd(2-aba)(NO_3_)(4-bphz)_3/2_]_n_·n(dmf) (**1**), [Cd(2-aba)_2_(4-bphz)]_n_·0.75n(dmf) (**2**), [Cd(seb)(4-bphz)]_n_·n(H_2_O) (**3**), [Cd(seb)(4-bpmhz)]_n_·n(H_2_O) (**4**), [Cd(hpa)(3-bphz)]_n_ (**5**), [Zn(1,3-bdc)(3-bpmhz)]_n_·n(MeOH) (**6**), [Cd(1,3-bdc)(3-bpmhz)]_n_·0.5n(H_2_O)·0.5n(EtOH) (**7**) together with the 2D coordination polymer [Cd(NO_3_)_2_(3-bphz)(bpe)]_n_·n(3-bphz) (**8**). The five topologically similar 2D networks **3**–**7** are built in the same way from a ladder-type chain based on [M_2_(CO_2_)_4_] metal building blocks interconnected by double pillars of azine ligands and double cross-linked by dicarboxylate ligands. The decisive role of the second carboxylic group in structure extension is evident from the double-chain 1D coordination polymer **2** together with structural analogs reported earlier [26], which have all been obtained with the use of monocarboxylic acids and look like structural precursors for the 2D networks. The 2D networks accommodate solvents in the interlayer space. The favorable criss-cross packing of the 1D coordination polymer **2** accompanied by the confinement of dmf solvent in the pores allowed tracing the solvent-exchange processes, which occurred without degradation of a coordination backbone. The ligand-based emission for all compounds was registered. In the reported cases, the desolvated forms reveal an essential increase in the emission intensity and can be suggested as sensors for small molecules. Since the main intensity of emission for all samples is concentrated in the range of 500–600 nm, they may be considered as useful materials for making physiologically-friendly yellow and yellow-green lighting sources.

## Supplementary Materials

The following data are available, Table S1: Bond lengths and angles for **1**–**8**, Figure S1: Infrared spectra of compounds **1**–**8**, Figure S2: XRPD patterns of compound **2** and its modified forms, Figure S3: XRPD patterns of compound **7**, Figure S4: N_2_ adsorption-desorption isotherms for desolvated compounds **2d** and **7d** at 77 K. Table S2: Crystal data and structure refinement parameters for **1**–**8**.
