# Supplementary material for: From 1D to 2D Cd(II) and Zn(II) Coordination Networks by Replacing Monocarboxylate with Dicarboxylates in Partnership with Azine Ligands: Synthesis, Crystal Structures, Inclusion, and Emission Properties
Source: Molecules. 2020 Nov 29;25(23):5616. doi: 10.3390/molecules25235616 (PMC7730819; doi:10.3390/molecules25235616)
Supplement: Supplementary file 1 [file molecules-25-05616-s001.pdf]

Supporting Information for the manuscript

From 1D to 2D Cd(II) and Zn(II) Coordination Networks by Replacing Monocarboxylate with Dicarboxylates in Partnership with Azine Ligands: Synthesis, Crystal Structures, Inclusion, and Emission Properties

Victor Ch.KRAVTSOV[a], Vasile LOZOVAN[b,c], Nikita SIMINEL[a], Eduard B. COROPCEANU[b,c], Olga V. KULIKOVA[a], Natalia V. COSTRIUCOVA[a], Marina S. FONARI[a,*]

[c]Institute of Applied Physics, Academiei 5, MD2028, Chisinau, Republic of Moldova
E-mails: kravtsov@phys.asm.md; siminel.n@gmail.com; olga.kulikova@phys.asm.md; costriucova@phys.asm.md; fonari.xray@phys.asm.md
[b]Institute of Chemistry, Academiei 3, MD2028, Chisinau, Republic of Moldova
[c]Tiraspol State University, Chemistry Department, Iablocikin 5, MD2069, Chisinau, Republic of Moldova
E-mails: lozovanvasile90@gmail.com; coropceanu.eduard@ust.md;

Table S1. Bond lengths [Å] and angles [°] for 1-8

**1**			
Cd(1)-N(5)#1	2.333(3)	Cd(1)-O(2)	2.356(3)
Cd(1)-N(2)	2.340(3)	Cd(1)-N(6)	2.391(3)
Cd(1)-O(1)	2.348(3)	Cd(1)-O(3)	2.398(3)
N(5)#1-Cd(1)-N(2)	169.36(10)	O(1)-Cd(1)-N(6)	141.55(10)
N(5)#1-Cd(1)-O(1)	89.61(9)	O(2)-Cd(1)-N(6)	86.24(10)
N(2)-Cd(1)-O(1)	89.90(10)	N(5)#1-Cd(1)-O(3)	87.41(9)
N(5)#1-Cd(1)-O(2)	94.48(10)	N(2)-Cd(1)-O(3)	85.44(11)
N(2)-Cd(1)-O(2)	93.98(11)	O(1)-Cd(1)-O(3)	135.11(9)
O(1)-Cd(1)-O(2)	55.32(9)	O(2)-Cd(1)-O(3)	169.49(9)
N(5)#1-Cd(1)-N(6)	93.39(10)	N(6)-Cd(1)-O(3)	83.33(10)
N(2)-Cd(1)-N(6)	93.60(10)		
Symmetry transformations used to generate equivalent atoms: #1 *x*, *y*, *z*-1			
**2**			
Cd(1)-N(3)	2.303(6)	Cd(2)-N(9)	2.322(5)
Cd(1)-N(6)#1	2.316(6)	Cd(2)-O(8)	2.332(6)
Cd(1)-O(1)	2.316(5)	Cd(2)-O(5)	2.336(5)

Cd(1)-O(3)	2.323(5)	Cd(2)-N(10)	2.354(6)
Cd(1)-O(4)#2	2.378(5)	Cd(2)-O(6)#3	2.416(6)
Cd(1)-O(2)	2.465(6)	Cd(2)-O(7)	2.448(7)
Cd(1)-O(4)	2.580(5)	Cd(2)-O(6)	2.542(6)
N(3)-Cd(1)-N(6)#1	174.4(2)	N(9)-Cd(2)-O(8)	97.3(2)
N(3)-Cd(1)-O(1)	93.5(2)	N(9)-Cd(2)-O(5)	92.8(2)
N(6)#1-Cd(1)-O(1)	89.9(2)	O(8)-Cd(2)-O(5)	140.3(2)
N(3)-Cd(1)-O(3)	90.2(2)	N(9)-Cd(2)-N(10)	173.5(2)
N(6)#1-Cd(1)-O(3)	90.1(2)	O(8)-Cd(2)-N(10)	83.6(2)
O(1)-Cd(1)-O(3)	139.5(2)	O(5)-Cd(2)-N(10)	90.5(2)
N(3)-Cd(1)-O(4)#2	85.4(2)	N(9)-Cd(2)-O(6)#3	84.9(2)
N(6)#1-Cd(1)-O(4)#2	90.1(2)	O(8)-Cd(2)-O(6)#3	89.4(2)
O(1)-Cd(1)-O(4)#2	91.62(19)	O(5)-Cd(2)-O(6)#3	129.8(2)
O(3)-Cd(1)-O(4)#2	128.88(19)	N(10)-Cd(2)-O(6)#3	88.7(2)
O(2)-Cd(1)-O(4)	137.41(18)	N(9)-Cd(2)-O(7)	94.0(2)
N(3)-Cd(1)-O(2)	91.7(2)	O(8)-Cd(2)-O(7)	54.0(2)
N(6)#1-Cd(1)-O(2)	93.9(2)	O(5)-Cd(2)-O(7)	87.2(2)
O(1)-Cd(1)-O(2)	53.86(19)	N(10)-Cd(2)-O(7)	91.7(2)
O(3)-Cd(1)-O(2)	85.75(19)	O(6)#3-Cd(2)-O(7)	143.0(2)
O(4)#2-Cd(1)-O(2)	145.17(19)	N(9)-Cd(2)-O(6)	86.58(19)
N(3)-Cd(1)-O(4)	81.45(19)	O(8)-Cd(2)-O(6)	165.2(2)
N(6)#1-Cd(1)-O(4)	94.33(19)	O(5)-Cd(2)-O(6)	53.12(19)
O(1)-Cd(1)-O(4)	167.37(19)	N(10)-Cd(2)-O(6)	91.0(2)
O(3)-Cd(1)-O(4)	52.54(18)	O(6)#3-Cd(2)-O(6)	76.70(19)
O(4)#2-Cd(1)-O(4)	76.49(18)	O(7)-Cd(2)-O(6)	140.3(2)
Symmetry transformations used to generate equivalent atoms: #1 *x*, *y*-1, *z*; #2 2-*x*, -*y*, 1-*z*; #3 1-*x*, 1-*y*, 2-*z*			
**3**			
Cd(1)-O(3)#1	2.331(4)	Cd(1)-O(1)	2.384(4)
Cd(1)-N(4)#2	2.336(4)	Cd(1)-O(4)#3	2.416(3)
Cd(1)-N(1)	2.348(4)	Cd(1)-O(3)#3	2.466(4)
Cd(1)-O(2)	2.361(4)		
O(3)#1-Cd(1)-N(4)#2	89.57(13)	O(2)-Cd(1)-O(1)	54.84(13)
O(3)#1-Cd(1)-N(1)	89.92(13)	O(3)#1-Cd(1)-O(4)#3	126.27(14)
N(4)#2-Cd(1)-N(1)	178.45(14)	N(4)#2-Cd(1)-O(4)#3	90.22(12)
O(3)#1-Cd(1)-O(2)	144.94(13)	N(1)-Cd(1)-O(4)#3	88.89(12)
N(4)#2-Cd(1)-O(2)	96.75(14)	O(2)-Cd(1)-O(4)#3	88.29(14)
N(1)-Cd(1)-O(2)	84.50(14)	O(1)-Cd(1)-O(4)#3	142.98(15)
O(3)#1-Cd(1)-O(1)	90.74(14)	O(3)#1-Cd(1)-O(3)#3	73.76(13)
N(4)#2-Cd(1)-O(1)	90.98(13)	N(4)#2-Cd(1)-O(3)#3	89.40(13)
N(1)-Cd(1)-O(1)	90.49(13)	N(1)-Cd(1)-O(3)#3	89.05(13)
O(2)-Cd(1)-O(3)#3	140.42(12)	O(1)-Cd(1)-O(3)#3	164.50(13)

O(4)#3-Cd(1)-O(3)#3	52.50(12)		
Symmetry transformations used to generate equivalent atoms: #1 2-*x*, 1-*y*, 1-*z*; #2 *x*+1, *y*, *z*+1; #3 *x*, *y*, *z*+1			
**4**			
Cd(1)-O(3)#3	2.344(2)	Cd(1)-O(1)#1	2.310(2)
Cd(1)-N(4)#2	2.342(2)	Cd(1)-O(4)	2.3900(19)
Cd(1)-N(1)	2.323(2)	Cd(1)-O(3)	2.456(2)
Cd(1)-O(2)#1	2.485(2)		
O(1)#1-Cd(1)-N(1)	92.75(8)	O(3)#3-Cd(1)-O(4)	126.13(7)
O(1)#1-Cd(1)-N(4)#2	88.09(8)	O(1)#1-Cd(1)-O(3)	161.61(8)
N(1)-Cd(1)-N(4)#2	177.37(8)	N(1)-Cd(1)-O(3)	95.75(8)
O(1)#1-Cd(1)-O(3)#3	90.63(8)	N(4)#2-Cd(1)-O(3)	82.75(8)
N(1)-Cd(1)-O(3)#3	90.00(8)	O(3)#3-Cd(1)-O(3)	73.12(8)
N(4)#2-Cd(1)-O(3)#3	87.50(8)	O(4)-Cd(1)-O(3)	53.29(7)
O(1)#1-Cd(1)-O(4)	143.12(8)	O(1)#1-Cd(1)-O(2)#1	54.27(8)
N(1)-Cd(1)-O(4)	90.24(8)	N(1)-Cd(1)-O(2)#1	101.33(8)
N(4)#2-Cd(1)-O(4)	90.57(8)	N(4)#2-Cd(1)-O(2)#1	81.18(8)
O(3)#3-Cd(1)-O(2)#1	143.17(8)	O(4)-Cd(1)-O(2)#1	89.12(8)
O(3)-Cd(1)-O(2)#1	138.74(7)		
Symmetry transformations used to generate equivalent atoms: #1 *x*, *y*, *z*+1; #2 *x*, *y*+1, *z*+1; #3 1-*x*, 1-*y*, 2-*z*			
**5**			
Cd(1)-O(3)#1	2.232(2)	Cd(1)-N(4)#3	2.322(3)
Cd(1)-O(4)#2	2.245(2)	Cd(1)-N(1)	2.335(3)
Cd(1)-O(1)	2.296(2)	Cd(1)-O(2)	2.503(2)
O(3)#1-Cd(1)-O(4)#2	124.06(9)	O(1)-Cd(1)-N(1)	89.61(9)
O(3)#1-Cd(1)-O(1)	93.07(8)	N(4)#3-Cd(1)-N(1)	172.18(8)
O(4)#2-Cd(1)-O(1)	142.61(8)	O(3)#1-Cd(1)-O(2)	146.04(8)
O(3)#1-Cd(1)-N(4)#3	87.41(9)	O(4)#2-Cd(1)-O(2)	88.46(8)
O(4)#2-Cd(1)-N(4)#3	83.39(9)	O(1)-Cd(1)-O(2)	54.21(7)
O(1)-Cd(1)-N(4)#3	95.30(9)	N(4)#3-Cd(1)-O(2)	87.04(9)
O(3)#1-Cd(1)-N(1)	98.42(8)	N(1)-Cd(1)-O(2)	90.93(9)
O(4)#2-Cd(1)-N(1)	89.01(9)		
Symmetry transformations used to generate equivalent atoms: #1 1-*x*, -*y*, 2-*z*; #2 *x*+1, *y*, *z*; #3 *x*+1, *y*, *z*+1			
**6**			
Zn(1)-O(3)#1	2.0246(16)	Zn(1)-N(1)	2.163(2)
Zn(1)-O(4)#2	2.0538(16)	Zn(1)-N(4)#3	2.164(2)
Zn(1)-O(2)	2.1446(17)	Zn(1)-O(1)	2.3165(19)
O(3)#1-Zn(1)-O(4)#2	117.42(7)	O(2)-Zn(1)-N(4)#3	91.71(8)
O(3)#1-Zn(1)-O(2)	152.02(7)	N(1)-Zn(1)-N(4)#3	176.76(7)
O(4)#2-Zn(1)-O(2)	90.32(7)	O(3)#1-Zn(1)-O(1)	94.06(7)

O(3)#1-Zn(1)-N(1)	89.79(8)	O(4)#2-Zn(1)-O(1)	148.43(7)
O(4)#2-Zn(1)-N(1)	86.59(8)	O(2)-Zn(1)-O(1)	58.11(6)
O(2)-Zn(1)-N(1)	88.26(8)	N(1)-Zn(1)-O(1)	91.49(8)
O(3)#1-Zn(1)-N(4)#3	91.75(8)	N(4)#3-Zn(1)-O(1)	91.24(8)
O(4)#2-Zn(1)-N(4)#3	90.17(8)		
Symmetry transformations used to generate equivalent atoms #1 *x, y*+1, *z;* #2 -*x*, 2-*y*, 2-*z*; #3 *x*, *y*+1, *z*+1			
**7**			
Cd(1)-O(2)#1	2.225(3)	Cd(1)-N(4)#3	2.33(3)
Cd(1)-O(1)#2	2.278(4)	Cd(1)-O(4)	2.380(4)
Cd(1)-N(1)	2.307(5)	Cd(1)-O(3)	2.390(13)
O(2)#1-Cd(1)-O(1)#2	122.27(14)	O(2)#1-Cd(1)-O(4)	91.86(13)
O(2)#1-Cd(1)-N(1)	89.7(2)	O(1)#2-Cd(1)-O(4)	144.95(13)
O(1)#2-Cd(1)-N(1)	86.5(2)	N(1)-Cd(1)-O(4)	102.7(2)
O(2)#1-Cd(1)-N(4)#3	84(2)	N(4)#3-Cd(1)-O(4)	87(2)
O(1)#2-Cd(1)-N(4)#3	88(2)	O(2)#1-Cd(1)-O(3)	141.7(3)
N(1)-Cd(1)-N(4)#3	168(2)	O(1)#2-Cd(1)-O(3)	93.5(3)
N(1)-Cd(1)-O(3)	107.2(3)	N(4)#3-Cd(1)-O(3)	84(2)
O(4)-Cd(1)-O(3)	51.4(3)		
Symmetry transformations used to generate equivalent atoms: #1 *x*, *y*-1, *z*; #2 1-*x*, 1-*y*, 1-*z*; #3 *x*-1, *y*, *z*+1			
**8**			
Cd(1)-N(1)	2.337(2)	Cd(1)-O(1)	2.326(4)
Cd(1)-N(3)	2.363(2)		
N(1)-Cd(1)-N(3)	93.94(8)	N(1)#1-Cd(1)-O(1)	90.36(13)
N(1)#1-Cd(1)-N(3)	86.06(8)	N(3)#1-Cd(1)-O(1)	100.58(15)
N(1)-Cd(1)-O(1)	89.64(13)	N(3)-Cd(1)-O(1)	79.42(15)
Symmetry transformations used to generate equivalent atoms: #1 2-*x*, 2-*y*, 1-*z*			

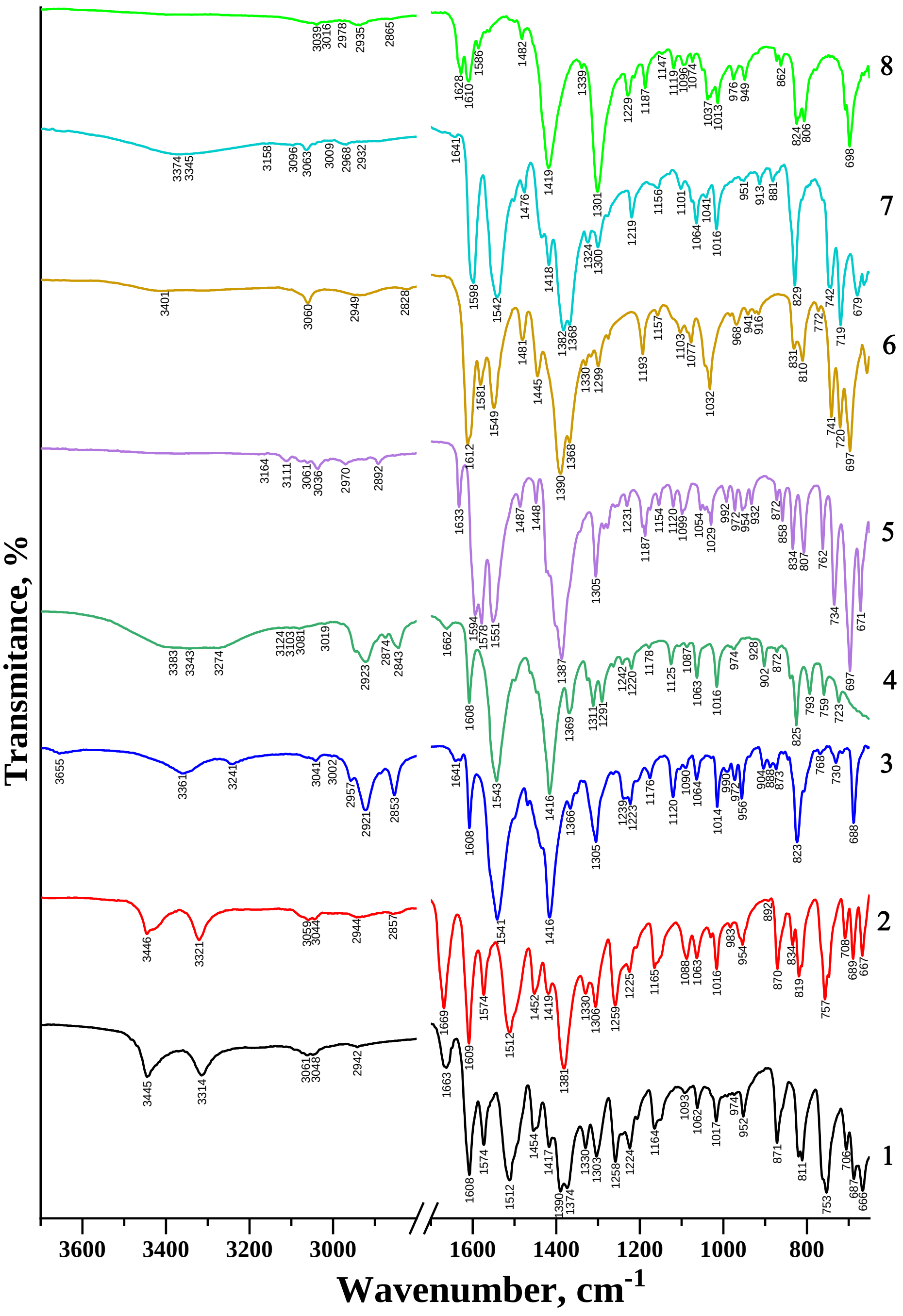

Figure S1. Infrared spectra of compounds **1-8**

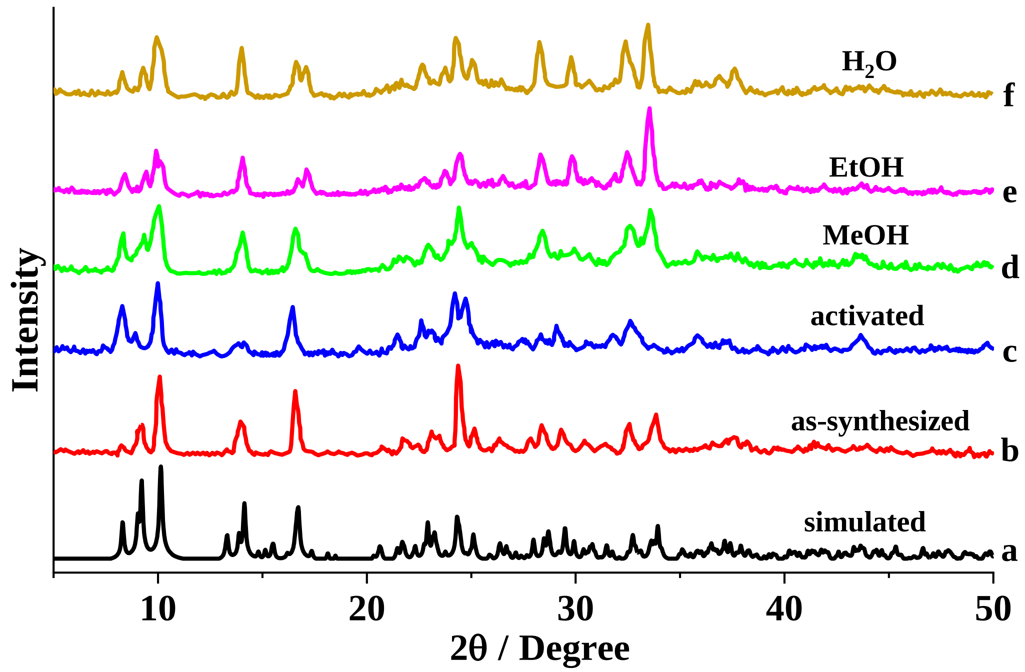

Figure S2. XRPD patterns for compound **2**: (a) simulated; (b) as-synthesized; (c) desolvated product; (d) product after keeping the crystals in MeOH solvent; (e) product after keeping the crystals in EtOH solvent; (f) product after keeping the crystals in H_2O solvent.

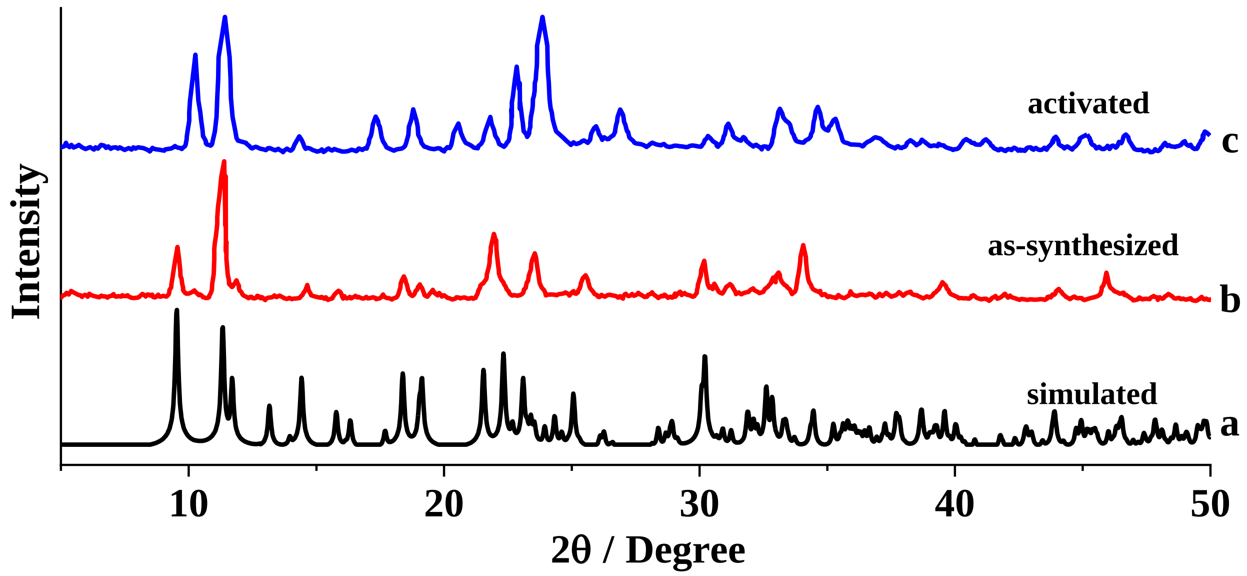

Figure S3. XRPD patterns of compound **7**: (a) simulated; (b) as-synthesized; (c) desolvated product.

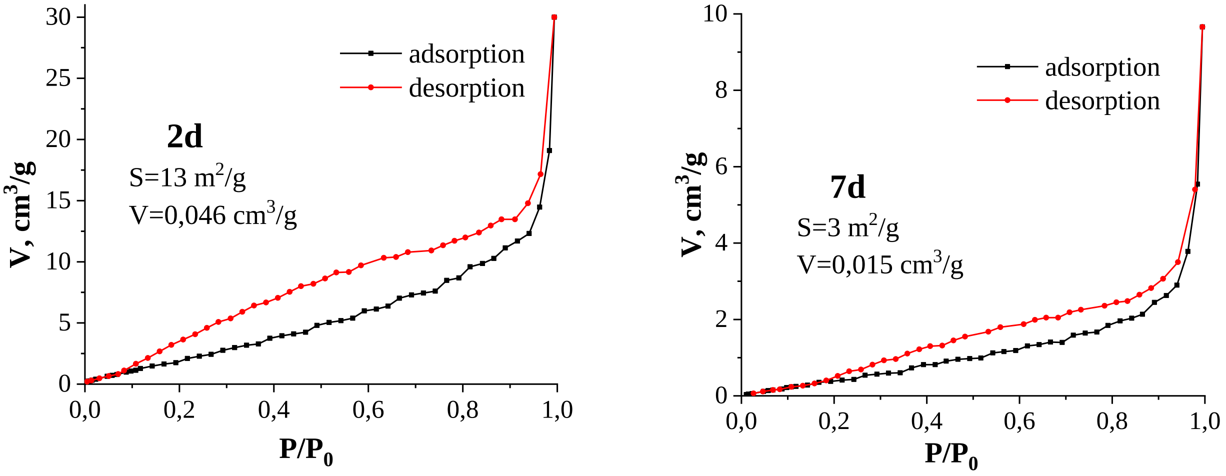

Figure S4. N_2 adsorption-desorption isotherms for desolvated compounds **2d** and **7d** at 77K.

Table S2. Crystal data and structure refinement parameters for **1–8**.

**Compound**	**1**	**2**	**3**	**4**	**5**	**6**	**7**	**8**
CCDC number	2022947	2022948	2022949	2022950	2022951	2022952	2022953	2022954
Empirical formula	$C_{28}H_{27}CdN_9O_6$	$C_{28.25}H_{27.25}CdN_{6.75}O_{4.75}$	$C_{22}H_{28}CdN_4O_5$	$C_{24}H_{32}CdN_4O_5$	$C_{21}H_{16}CdN_4O_4$	$C_{23}H_{22}N_4O_5Zn$	$C_{23}H_{22}CdN_4O_5$	$C_{36}H_{32}CdN_{12}O_6$
Formula weight	697.98	649.72	540.88	568.93	500.78	499.81	546.84	841.13
Crystal system	Triclinic	Triclinic	Triclinic	Triclinic	Triclinic	Triclinic	Triclinic	Triclinic
Space group	*P*-1	*P*-1	*P*-1	*P*-1	*P*-1	*P*-1	*P*-1	*P*-1
a, Å	10.2003(7)	15.0892(8)	10.0768(8)	10.0126(6)	7.9286(5)	9.1741(5)	9.8636(6)	9.5960(7)
b, Å	10.3387(4)	15.8585(7)	10.1625(7)	11.3491(7)	10.2530(7)	10.0959(6)	10.3856(5)	10.0623(8)
c, Å	15.9371(10)	15.8843(7)	12.7653(7)	12.8116(8)	13.8228(7)	12.7739(6)	11.9413(8)	10.2938(8)
α, °	77.209(5)	60.650(5)	106.911(5)	97.992(5)	94.035(5)	103.514(5)	101.408(5)	66.957(8)
β, °	88.096(5)	65.928(5)	91.992(5)	97.620(5)	100.886(5)	95.606(4)	92.074(5)	88.604(7)
γ, °	84.390(5)	65.649(5)	109.708(7)	114.365(6)	111.823(6)	104.631(5)	104.721(5)	87.270(7)
Volume, $Å^3$	1631.03(17)	2915.2(3)	1164.57(15)	1283.84(15)	1012.12(12)	1097.52(11)	1154.90(13)	913.58(14)
Z	2	4	2	2	2	2	2	1
D(calcd) Mg/m^3	1.421	1.479	1.542	1.472	1.643	1.512	1.573	1.529
μ, mm^{-1}	0.717	0.797	0.977	0.891	1.114	1.162	0.987	0.662
F(000)	708	1318	552	584	500	516	552	428
Reflections collected	10,619	18,829	6319	8185	5443	7355	7156	4900
Independent reflections	5723 [*R*(int) = 0.0378]	10,524 [*R*(int) = 0.0354]	4061 [*R*(int) = 0.0328]	4767 [*R*(int) = 0.0296]	3563 [*R*(int) = 0.0230]	4062 [*R*(int) = 0.0263]	4281 [*R*(int) = 0.0319]	3196 [*R*(int) = 0.0215]
Reflections with [$I > 2\sigma(I)$]	4776	7264	3182	4057	3126	3428	3619	2927
Data/restraints/parameters	5723/72/431	10,521/320/833	4061/3/322	4767/0/310	3563/0/271	4062/38/323	4281/97/436	3196/24/287
Goodness-of-fit on F^2	0.991	1.002	1.009	1.039	1.008	1.077	1.000	1.030
Final *R* indices [$I > 2\sigma(I)$], R_1, wR_2	0.0404, 0.0852	0.0612, 0.1640	0.0461, 0.0813	0.0340, 0.0669	0.0322, 0.0575	0.0370, 0.0839	0.0446, 0.1061	0.0338, 0.0721
R indices (all data) R_1, wR_2	0.0506, 0.0915	0.0958, 0.1857	0.0649, 0.0907	0.0438, 0.0708	0.0397, 0.0615	0.0480, 0.0898	0.0562, 0.1141	0.0395, 0.0756
Largest diff. peak and hole e. $Å^{-3}$	0.735 and −0.476	0.843 and −0.670	0.789 and −0.496	0.489 and −0.460	0.441 and −0.483	0.330 and −0.255	0.710 and −0.501	0.396 and −0.431
